# Induced Pluripotent Stem Cells of Microtus levis x Microtus arvalis Vole Hybrids: Conditions Necessary for Their Generation and Self-Renewal

**Published:** 2015

**Authors:** E. V. Grigor’eva, A. I. Shevchenko, S. P. Medvedev, N. A. Mazurok, A. I. Zhelezova, S. M. Zakian

**Affiliations:** Federal Research Center, Institute of Cytology and Genetics, Siberian Branch of the Russian Academy of Sciences, Lavrentiev Ave., 10, 630090, Novosibirsk, Russia; Institute of Chemical Biology and Fundamental Medicine, Siberian Branch of the Russian Academy of Sciences, Lavrentiev Ave., 8, 630090, Novosibirsk, Russia; State Research Institute of Circulation Pathology, Ministry of Healthcare of the, Rechkunovskaya Str., 15, 630055, Novosibirsk, Russia; Novosibirsk State University, Pirogova Str., 2, 630090, Novosibirsk, Russia

**Keywords:** reprogramming, induced pluripotent stem cells, common voles

## Abstract

Every year, the list of mammalian species for which cultures of pluripotent stem cells (PSCs) are generated increases. PSCs are a unique tool for extending the limits of experimental studies and modeling different biological processes. In this work, induced pluripotent stem cells (iPSCs) from the hybrids of common voles *Microtus levis *and *Microtus arvalis*, which are used as model objects to study genome organization on the molecular-genetic level and the mechanisms of X-chromosome inactivation, have been generated. Vole iPSCs were isolated and cultured in a medium containing cytokine LIF, basic fibroblast growth factor (bFGF), ascorbic acid, and fetal bovine serum. Undifferentiated state of vole iPSCs is maintained by activation of their endogenous pluripotency genes – *Nanog*, *Oct4*, *Sox2*, *Sall4, *and *Esrrb*. The cells were able to maintain undifferentiated state for at least 28 passages without change in their morphology and give rise to three germ layers (ectoderm, mesoderm and endoderm) upon differentiation.

## INTRODUCTION

Currently, in addition to the traditional method of isolating pluripotent stem cells (PSCs) from early mammalian embryos, it has also become possible to induce pluripotency by reprogramming different types of terminally differentiated somatic cells [1-4]. Reprogramming of somatic cells to a pluripotent state allows one to obtain an unlimited amount of autologous iPSCs of any mammal, including humans. Reprogramming technology holds tremendous prospects not only for a personalized approach to the treatment of various diseases, but also serves as a tool for genetic modeling of many biological processes, including the study of early embryonic development, the signaling pathways and factors involved in pluripotency maintenance, and the triggering of differentiation.

Reprogramming of differentiated cells of various mammalian species to a pluripotent state is possible due to the overexpression of four transcription factors. These factors are Oct4, Sox2, Klf4, and c-Myc (OSKM). The genes are evolutionarily conserved in mammals [5, 6], and their products have a substantially overlapping set of key target genes, and thus genetic constructs expressing human or mouse OSKM can be frequently applied for cell reprogramming in different mammalian species [7-10]. To date, iPSCs of mouse, human, macaque, rat, dog, many farm animals and some other mammalian species, including prairie vole* Microtus ochrogaster*, have been obtained. The conditions for induction and maintenance of pluripotency vary among the species [11-19]. This is partly due to the species-specificity of the signaling cascades involved in the activation and maintenance of an undifferentiated state *in vitro*, and due to various requirements for the composition of the culture medium, e.g. the presence or absence of bovine serum in the medium. Induction and maintenance of pluripotency are facilitated by the presence in the medium of inhibitors of various signaling pathways, inhibitors of histone deacetylases, histone and DNA methyltransferases, as well as antioxidants.

In the current study, the conditions for obtaining and maintaining cells with induced pluripotency of the common vole species of genus *Microtus *have been selected. Four closely related cryptic species, *M. arvalis*,* M. levis*, *M. transcaspicus, *and *M. kirgisorum, *comprising a group of common voles are the subject of a molecular genetic study of genome organization and the mechanisms of X chromosome inactivation [20-28]. The genes involved in the establishment and maintenance of pluripotency in these species have been studied, and their conservation has been demonstrated, including the expression pattern [5, 6, 29]. The existence of pluripotent cells of common voles might become an appropriate tool for molecular-genetic studies of these species.

Previously, we have undertaken numerous attempts to obtain PSCs of common vole species of the genus* Microtus *from early pre-implantation blastocysts and germinal cells [30]. Multipotent cell lines of the pre-implantation embryo, such as trophoblast stem cells and extraembryonic endoderm cells, have been derived [25, 31-33]. However, PSCs of common voles have not been obtained yet. In the experiments on somatic cell reprogramming presented in this work, we managed for the first time to determine the conditions that allow one to obtain and maintain PSCs of common voles.

## MATERIALS AND METHODS

**Cell cultures and media**

Skin fibroblasts and brain cells were isolated from* M. levis × M. arvalis *hybrid embryos on day 19 (E19) of embryonic development and then cultured. The fibroblasts were grown in DMEM/F12 (F12, Nutrient Mixture/Dullbecco’s Modified Eagle Medium; Gibco) 1:1 supplemented with 10% fetal bovine serum (FBS; Gibco), 1× Non-Essential Amino Acids (NEAA; Gibco), 1× Pen Strep (100 u/ml penicillin, 100 μg/ml streptomycin; Gibco), 1× GlutaMAX (Gibco). Brain cells were grown in DMEM/F12 (1:1) with 10% FBS, 1× NEAA, 1× Pen Strep, 1× GlutaMAX for the first 3 days, then transferred to Schneider’s medium: DMEM/F12 (1:1), 10 ng/ml bFGF (StemCell), 10 ng/ml EGF (Sigma), 2 μg/ml heparin (Sigma), 1× N2 Supplement (Gibco), 1× Pen Strep, 1× GlutaMAX.

In the first experiment, two media were used for the induction of pluripotency in *M. levis *× *M. arvalis* hybrid cells, which were previously used to obtain* M. ochrogaster *iPSCs [16]*. *The media were prepared from Advanced DMEM/F12 (1:1) (Gibco) containing 15% KSR (Knockout Serum Replacement; Gibco), 1× NEAA, 0.1 mM 2-mercaptoethanol, 1× Pen Strep, and 1× GlutaMAX. The first medium was supplemented with three inhibitors of signaling pathways (3iR conditions): CHIR99021 (3 μM, StemRD), PD0325901 (1 μM, StemRD), A83-01 (0.5 μM, Stemgent), and ROCK inhibitor (Y-27632, 10 μM, StemRD); the second medium did not contain any inhibitor. Both media contained 1,000 u/ml mouse LIF (mLIF, Invitrogen) and 2 μg/ml doxycycline (Sigma), which was added only at the initial stages of cultivation. Mouse embryonic fibroblasts mitotically inactivated by a mitomycin C solution (10 mg/ml, Sigma) for 2 h were used as a feeder substrate for the generation and cultivation of vole iPSCs. Transfer of individual primary colonies at the first passage was performed with a glass capillary, and further using TrypLE (Gibco).

In further experiments on reprogramming in the medium based on Advanced DMEM/F12 (1:1) supplemented with 1× NEAA, 0.1 mM 2-mercaptoethanol, 1× Pen Strep, and 1× GlutaMAX, we varied the composition of 3iR, mLIF, PKCi (Go6983, Tocris, 5 μM), bFGF, ascorbic acid, KSR, and FBS. iPSCs of hybrid *M. levis × M. arvalis *were derived in the medium, which included: Advanced DMEM/F12 (1 : 1), 7% KSR (Gibco), 7% FBS (Gibco), 0.1 mM 2-mercaptoethanol, 1× Pen Strep, 1× GlutaMAX, 1000 u/ml mLIF (Invitrogen), 10 ng/ml bFGF (StemCell), 50 mkg/ml ascorbic acid (Wako). The medium initially contained 2 μg/ml doxycycline (Sigma), which was abrogated on day 14 of reprogramming. Vole iPSCs were frozen in 90% FBS and 10% DMSO.

**Plasmid constructs, generation of lentiviruses, transduction scheme**

Three plasmids were used in the experiments: 1) TetO- FUW-OSKM (Addgene Plasmid 20321) encoding mouse reprogramming factors OSKM; 2) FUdeltaGWrtTA (Addgene Plasmid 19780) carrying tetracycline transactivator cDNA necessary for regulation of the transcriptional activity of the construct with OSKM by supplementing the growth media with doxycycline; 3) pGpur expressing a green fluorescent protein (EGFP) gene for monitoring transduction efficiency. Lentiviruses were obtained by cotransfection of the plasmids into 293FT cells with pxPAX2 (Addgene Plasmid 12260) and pMD2.G (Addgene Plasmid 12259) plasmids encoding the proteins required for packaging of viral particles [34, 35]. Human embryo kidney cells, 293FT, were seeded at a density of 6 × 10^6^ into T75 culture flasks and incubated overnight. Transfection was performed by the calcium phosphate method [36]. The medium with viral particles was harvested and filtered (0.45 μm; Millipore) 48, 72, and 96 h after transfection. Viruses were concentrated using an ultracentrifuge (Beckman Coulter, Optima XE-90 Ultracentrifuge) for 90 min at 70, 000 *g*. A viral pellet was dissolved in 200 μl of F12/DMEM and kept in aliquots at -70oC.

The fibroblasts and cells isolated from the brain were seeded in 12-well plates at a density of 50 × 10^3^ to 75 × 10^3^ cells per well, respectively, 24 h before transduction. The cells were transduced for 4-6 passages. On the day of transduction, the medium with lentiviruses obtained using TetO-FUW-OSKM, FUdeltaGW-rtTA, or pGpur plasmids was added for 14-16 h, with a titer of about 3 × 10^7^ infectious units per 1 ml (MOI 5-7.5) for each of the lentiviruses and 4 μg/ml polybrene (Hexadimethrine bromide, Sigma). After 4 days, cells transduced by lentiviruses with reprogramming factors and tetracycline transactivator were passaged using TrypLE at a dilution of 1:10 to 1:20 (depending on cell density in a well) on mitotically inactivated mouse embryonic fibroblasts in culture media varying in composition. For determination of transduction efficiency, cells transduced with lentiviruses containing pGpur were used, and the assessment of the percentage of green cells was performed using a fluorescent microscope and/or flow cytometry 4 days after transduction.

**Histochemical detection of endogenic alkaline phosphatase activity**

The activity of endogenic alkaline phosphatase (AP) was measured histochemically according to [37]. Cells were fixed by air-drying and incubated in a dye solution: 100 μM Tris-HCl pH 9.0; 100 μM NaCl; 5 μM MgCl_2_; 0.4 μg/ml naphthol phosphate (Sigma); 1 μg/ ml Fast Violet B Salt (Sigma) for 15–20 min in the dark at room temperature.

**Immunofluorescence assay**

Cells were fixed with 4% formaldehyde for 10-15 min at room temperature, washed with PBS, and incubated with 0.1% Triton X-100 and 2.5% FBS (or BSA) dissolved in PBS for 30 min at room temperature. Immunoprecipitation was performed by using primary antibodies overnight at 4oC. A list of the primary antibodies used in this study is provided in *Table 1*. Localization of primary antibodies was visualized using secondary anti-rabbit or -mouse antibodies conjugated to the fluorescent dyes Alexa 488 and Alexa 568 (Life Technologies). Nuclei were stained with DAPI (Vector Laboratories).

**Table 1:** List of the antibodies used in the study

Antibodies	Source	Catalogue number	Type	Working dilutions
SSEA1	Santa Cruz Biotechnology	sc-21702	mouse monoclonal IgM	1 : 50
OCT4	Santa Cruz Biotechnology	sc-5279	mouse monoclonal IgG2b	1 : 100
SOX2	Santa Cruz Biotechnology	sc-20088	rabbit polyclonal IgG	1 : 100
KLF4	Abcam	ab104846	mouse monoclonal IgG1	1 : 200
β-III- tubulin	Covance	MMS-435P-100	mouse monoclonal IgG2a	1 : 1000
Nestin	Abcam	ab6142	mouse monoclonal IgG1	1 : 400
α-SMA	Dako	M0851	mouse monoclonal IgG2a	1 : 100
CD90	Millipore	MAB1406	mouse monoclonal IgG2b	1 : 100
KRT18	Millipore	MAB3234	mouse monoclonal IgG	1 : 200
SOX17	Millipore	09-038	rabbit polyclonal IgG	1 : 100

***In vitro* differentiation of cell lines in embryoid bodies**

Colonies of undifferentiated cells were mechanically transferred onto Petri dishes coated with a thin layer of 1% agarose in DMEM/F12 (1:1) with 10% FBS, 1× Pen Strep and 1× GlutaMAX. The formed embryoid bodies were cultured for 7 days in suspension and transferred onto Petri dishes coated with 0.1% gelatin for attachment. Attached embryoid bodies were grown for 14-20 days, followed by analysis of differentiated cells by immunofluorescence assay or disaggregation using TrypLE with further RNA isolation and RT-PCR.

**RNA isolation, RT-PCR**

RNA was isolated with Trizol Reagent (Ambion). Samples were treated with DNase I (Turbo DNA-free, Ambion) in order to prevent DNA contamination; cDNA was synthesized using Super-ScriptIII (Invitrogen). Primer sequences and reaction conditions for RT-PCR are shown in *Table 2*. For the transcription analysis of the exogenous lentiviral cDNA of OSKM in mouse cells, we used the primers and RT-PCR conditions listed in [34]. A negative control reaction (RT-), with the reaction solution containing all the components necessary for cDNA synthesis, except for reverse transcriptase, was performed for every primer pair.

**Table 2:** Primers and PCR conditions

Gene	Nucleotide sequence	Primers	Mg2+ concentration, mM	Annealing temperature, °C
β-actin	gacggggtcacccacactgt gagtacttgcgctcaggaggag	β-actin-1 β-actin-2	3	60
Nanog	agtgtcttaaggacgcagaa atctcctaattgccaatacc	Nanog_QF1 Nanog_QR1	3	60
Oct4	ccaagctgctgaagcagaaga tttgaatgcatgggagagcccag	OCT4-2F OCT-5R	4	53
Sox2	tccatgaccagctcgcagacctac ccctcccaattcccttgtttctct	Sox2F Sox2R2	3	60
Sall4	tcaccaacgccgtcatgttacagc ggtgggctgtgctcggataaatgt	Sall4F Sall4R	2	60
Errβ	agctgcggctccttcatcaag cttgtacttctggcggcctcc	ERRB1F ERRB4R	1.5	63

**Bisulfite sequencing of vole Oct4 gene promoter DNA**

Bisulfite modification and purification of genomic DNA (500 ng) were conducted using EZ DNA Methylation – Direct Kit (Zymo Research). Modified DNA was further used for PCR with primers: Oct4_Reg2_f2 (5’-TAAGAGGATGGGGGGGTAGTGAAAG- 3’) Oct4_Reg2_r2 (5’-GAAATCTAAAACCAAATATCCAACCATAAA- 3’).

The obtained PCR products were cloned using pGEM-T Easy Vector System I (Promega). A total of 10 randomly selected plasmid clones of each DNA sample were sequenced using a universal M13 Reverse primer. Nucleotide sequences were analyzed with the QUMA software (Quantification tool for Methylation Analysis, http://quma.cdb.riken.jp/, [38]).

**Cytogenetic analysis**

Cells were fixed according to standard protocols [39] with several modifications: time of incubation with ethidium bromide (0.05%) – 3 h; with colcemid (10 μg/ml, Gibco) – 2 h; and hypotonic incubation – 15–20 min.

Prior to staining, samples were incubated overnight at 50oC. A Hoechst 33258 (Sigma) solution at a concentration of 0.05 μg/ml was prepared using Hank’s balanced solution (HBSS, Gibco). Metaphase spreads were stained in the Hoechst 33258 solution for 10 min, and then the samples were rinsed with water and placed in a acetate buffer (pH 5.5). The metaphase spreads were visualized using the microscope Ni-E (Nikon), Lucia software.

**Cell analysis by flow cytometry**

The number of EGFP- and SSEA1-positive cells was estimated using the BD FACS Aria and BD FACSCanto II systems with the BD FACS Diva software. Surface antigen SSEA1 was determined using antibodies (sc- 21702, Santa Cruz Biotechnology) according to the manufacturer’s protocol.

## RESULTS

**Obtaining doxycycline-dependant iPSClike vole cells and their characterization**

Common vole iPSCs were obtained using lentiviruses expressing cDNA of four key mouse reprogramming factors OSKM under a doxycycline-regulated promoter. Transcription from this promoter can be activated by adding an antibiotic in the culture medium; however, the cells should be previously transduced with lentiviruses expressing cDNA of a tetracycline-dependant transactivator. This system, where mouse OSKM expression is modulated by the addition of doxycycline, was successfully utilized previously for obtaining human and mouse iPSCs [34]. Mice and voles belong to the same family of rodents (Muridae), order Rodentia, and exhibit high similarity of OSKM genes [40]. The advantage of this system, in our opinion, is the fact that all four reprogramming factors are delivered into a cell by the same viral particle.

**Fig. 1:**
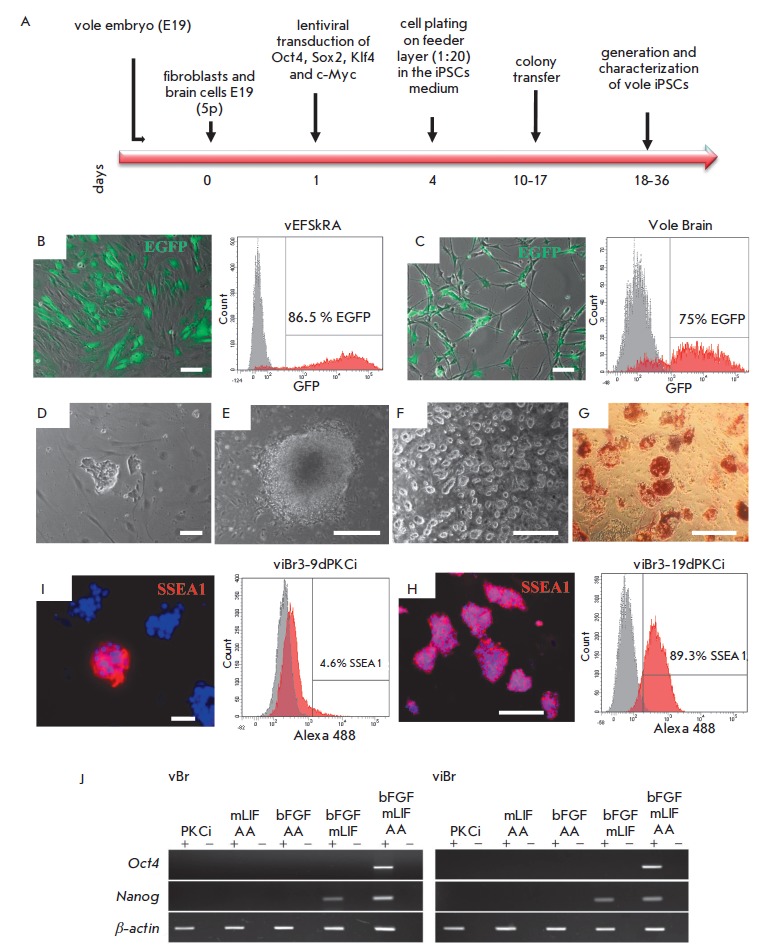
Obtaining and characterizing doxycycline-dependent iPSC-like lines of *M. levis *× *M. arvalis *hybrid cells.* A *– scheme of the experiment on obtaining iPSCs of the common vole of genus *Microtus*. *B, C *– assessment of the efficiency of the transduction of the embryonic skin fibroblasts (vEFSkRA cell line) and brain cells (Vole Brain) of hybrid voles with lentivirus expressing EGFP (green signal) by fluorescence microscopy and flow cytometry. The percentage of GFP-positive cells among the fibroblasts and brain cells is 86.5% and 75%, respectively. *D *– primary colony morphology on the 8th day of the reprogramming of cells isolated from the brain. *E *– colony morphology on the 13th day of reprogramming of cells isolated from the brain. *F *– morphology of viBr3 cell line colonies grown in the medium supplemented with mLIF + 3iR, passage 3. *G ***– **histochemical assay of endogenous AP activity in the viBr3 cell line, passage 2.* H, I ***– **data of immunofluorescence analysis and flow cytometry on the presence of SSEA1-positive cells in the viBr3 line on days 9 and 19 of culturing in the presence of PKCi, respectively. Nuclei are stained with DAPI (blue signal).* J ***– **RT-PCR analysis of *Oct4 *and *Nanog *expression in the vBr3 and viBr3 cell lines after culturing for 3 passages in the media supplemented with various components. AA – ascorbic acid. Scale bar *B–D, H *– 100 μm, *E–G, I *– 500 μm

Cells isolated from hybrid embryos of *M. levis *×* M. arvalis*, which are used as a model object for study ing the phenomenon of non-random inactivation of the X chromosome [27, 28], were transfected with lentiviral constructs. Two types of hybrid cells were used: skin fibroblasts and cells isolated from the brain. Transduction was performed according to the scheme depicted in *Fig. 1A*. Transduction efficiency was assessed using lentiviruses expressing a green fluorescent protein by flow cytometry, and fluorescent microscopy was 86.5% in fibroblasts (*Fig. 1C*) and 75% in brain cells (*Fig. 1B*).

Pluripotency in common vole cells was induced in two types of media that had previously been used for obtaining iPSCs from the fibroblasts of prairie vole* M. ochrogaster *[16]. The first type of media included Advanced DMEM/F12 with 15% KSR and mLIF, the second medium contained the same components, but it also included an inhibitor cocktail called 3iR. The cocktail 3iR contains inhibitors of kinases MEK/ERK and GSK3b (PD0325901 and CHIR99021, respectively), an antagonist of the IL-type receptor TGF-s (A83-01) used for obtaining mouse and rat PSCs and maintaining them in undifferentiated state, and a ROCK inhibitor that enhances the survival of single cells in culture [41-46].

Four days after transduction*, M. levis × M. arvalis* hybrid cells s were transferred on mitotically inactivated mouse embryonic fibroblasts and the medium with or without 3iR for obtaining iPSCs was added. The first morphological changes appeared 24 h after cell transfer, and an increase in cell proliferation was noted. After 5-8 days, the transduced cells that were isolated from the brain began to merge into primary homogeneous colonies (*Fig. 1D*), whereas the transduced fibroblasts did not give rise to such PSC-like primary colonies (*Table 3*).

**Table 3:** Conditions tested in experiments on obtaining iPSC lines of interspecies M. levis × M. arvalis vole hybrids

Cell type (experiment №)	Cell number, 10^3^	Transfection efficiency, %	Culturing conditions	Number of primary colonies of AP+ cells	Number of iPSC lines
Brain cells (I exp.)	37.5	75	KSR, mLIF	85	_
	37.5		KSR, mLIF, 3iR	200	_
Fibroblasts (I exp.)	25	86.5	KSR, mLIF	_	
	25		KSR, mLIF, 3iR	_	
Fibroblasts (II exp.)	12.5	32.9	KSR, mLIF, bFGF, AA	_	
	12.5		KSR, mLIF, bFGF, AA, 3iR	_	
	12.5		KSR, FBS, mLIF, bFGF, AA	70	2
	12.5		KSR, FBS, mLIF, bFGF, AA, 3iR	100	_

Note. AA – ascorbic acid.

Primary colonies obtained during brain cell reprogramming were transferred into individual wells by disaggregation with a glass capillary on day 10-13 after the start of the experiment. Cell morphology remained the same as in the case of mESCs: threedimensional dense colonies with tight intercellular contacts (*Fig. 1E,F*). The cells in these colonies had a high nuclear/cytoplasmic ratio, which is a characteristic of ESCs. After transfer of primary colonies, most cells underwent differentiation on the first passage. However, about 40% of the cells retained an ESC-like morphology. The difference in the level of endogenous AP was detected in selected colonies by histochemical staining (*Fig. 1G*). Cell lines with no detectable AP activity were excluded from further analysis. Thus, a total of 11 cell lines obtained in the presence of inhibitors (viBr; vole inhibitor Brain) and 10 lines obtained in their absence (vBr) were selected based on the results of an AP activity analysis at early passages. It should be noted that most of the cells did not express one of the major early markers of pluripotency specific to mouse PSCs, surface antigen SSEA1. The iPSC-like cells underwent rapid proliferation in the medium containing doxycycline without altering the morphology for approximately 40 passages (more than 120 days). The cells were passaged every 2-3 days at a dilution of 1:8 to 1:10: they resisted repeated freezing in liquid nitrogen with further thawing without changing their phenotypic characteristics.

We repeatedly tried to culture the cell lines in the absence of doxycycline, abolishing the expression of transgenic OSKM, but this led to the flattening of the plump colonies of iPSC-like cells and differentiation by the second day of growth.

Thus, culture conditions that allowed to successfully obtain *M. ochrogaster *iPSCs failed to induce pluripotency in *M. levis *× *M. arvalis *hybrid cells. When reprogramming in these media, the fibroblasts of common vole hybrids did not form even primary colonies and the cells isolated from the brain that nonetheless demonstrated primary colony formation, but yet did not show activation of self-renewal mechanisms, which would have enabled them to maintain an iPSC-like morphology in the absence of OSKM expression.

Further, we focused on the selection of culture medium components which would allow the induction and maintenance of pluripotency in common vole cells *in vitro*. In order to achieve this, we studied various combinations of such medium components as mLIF, PKCi, bFGF, and ascorbic acid, which are used for obtaining and maintaining pluripotent cell cultures of other mammalian species [7, 9, 13, 47-55]. Medium components were tested in two iPSC-like doxycycline-dependent lines, one of which had been obtained and grown in a medium supplemented with inhibitors (viBr3 line), and a medium without inhibitors (vBr3).

A peculiar effect was observed upon the addition of substance Go6983, a protein kinase C inhibitor (PKCi), to the medium, which is able to trigger and maintain the self-renewal capability of rodent pluripotent cells without activating LIF/STAT3- or suppressing ERK/ GSK3-signaling pathways [48, 50]. The appearance of 3–5% SSEA1-positive cells (*Fig. 1H*) was observed on the 9th day of culturing in both iPSCs-like cell lines in the presence of PKCi, and the percent of these cells increased to 80-90% by day 19 (*Fig. 1II*). However, despite the expression of SSEA1, one of the markers in mouse, rat and *M. ochrogaster *pluripotent cells cultured *in vitro* [16, 56, 57], iPSC-like cells of *M. levis *× *M. arvalis* hybrids still retained the ability to differentiate in the absence of doxycycline. A more significant for the reprogramming of common vole cells result has been obtained using a medium containing all of the three components: mLIF, bFGF, and ascorbic acid, which for the first time allowed us to grow iPSC-like cultures of *M. levis *× *M. arvalis *hybrids for six passages in the absence of doxycycline without visible changes in morphology. Activation of endogenous pluripotency genes* Oct4 *and *Nanog *expression has been noted in cell cultures (*Fig. 1J*). No similar effect has been shown upon addition of mLIF, bFGF, or ascorbic acid alone or in pairwise combinations to the culture medium for viBr3 and vBr3. However, since viBr3 and vBr3, which were cultured without doxycycline but in the presence of mLIF, bFGF, and ascorbic acid, showed differentiation after the sixth passage, we decided to perform the second experiment on reprogramming common vole cells, applying from the first stages the same conditions that allowed us to maintain for some period *M. levis* × *M. arvalis *hybrid iPSCs in the medium without doxycycline.

**Successful derivation of doxycyclineindependent vole iPSCs**

**Fig. 2:**
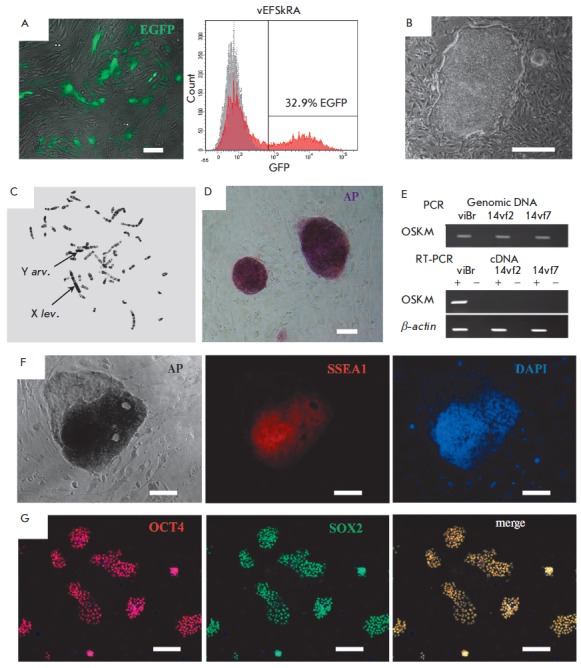
Obtaining and characterizing iPSCs of common vole *M. levis *× *M. arvalis *hybrids. *A *– efficiency of the transduction of vole embryonic fibroblasts (vEFSkRA) with a lentivirus expressing GFP (green signal), and assessment of the percentage of GFP-positive cells (32.9%) by fluorescence microscopy and flow cytometry. *B *–morphology of 14vf7 cell line colonies at passage 7. *C ***– **metaphase spread of 14vf7, passage 13. X *lev*. – X chromosome of *M. levis*, Y *arv*. – Y chromosome of *M. arvalis*. *D *– histochemical detection of endogenous AP activity, 14vf7 cell line, passage 6. *E ***–** RT-PCR analysis of the expression of the construct with exogenous factors of reprogramming (OSKM) in iPSC lines of common vole hybrids. *F *– immunofluorescence analysis of SSEA1 expression (red signal) and histochemical detection of AP activity, 14vf7 line, passage 4. Nuclei are stained with DAPI (blue signal). *G *– immunofluorescence analysis of pluripotency markers OCT4 (red signal) and SOX2 (green signal). Nuclei are stained with DAPI (blue signal). Scale bar *A, D, F, G *– 100 μm, *B *– 500 μm

New reprogramming of *M. levis *× *M. arvalis *hybrids was conducted according to the previously described scheme (*Fig. 1A*) using embryonic skin fibroblasts. The efficiency of transduction was 32.9% (*Fig. 2A*). Taking into account the experience gained in the previous experiment, fibroblasts were transferred to the medium for reprogramming containing mLIF, bFGF, and ascorbic acid after transduction with lentiviruses expressing OSKM and a tetracycline-dependent transactivator. As in the previous experiment, we used a medium with or without the 3iR cocktail. Furthermore, we varied the content of KSR and FBS in the media: some media contained 15% KSR as described above, whereas others included a mixture of 7% FBS and 7% KSR.

Primary colonies were observed in fibroblasts already by the third day of culturing (on the 7th day of transduction) in reprogramming media containing a mixture of FBS and KSR. The formation of primary colonies was not shown for the medium containing 15% KSR (*Table 3*). By day 10-14 from the start of transduction, six primary colonies from the plates with 3iR and eight colonies from the plates without 3iR had been transferred from the cultures reprogrammed in the presence of KSR and FBS. Doxycycline was canceled 14 days after the start of reprogramming, i.e. on the first passage after seeding primary colonies in individual wells. As a result of culturing without doxycycline, most of the colonies underwent differentiation or were excluded after the first analysis of AP activity. Thus, two clones expressing AP and growing in the medium without inhibitors, 14vf2 and 14vf7, were selected. Two cell lines obtained in this experiment were cultured in the medium containing 7% FBS, 7% KSR, mLIF, bFGF, and ascorbic acid without changes in morphology for at least 28 passages (more than 4 months) in the absence of doxycycline. Removal of any of the medium components, namely mLIF, bFGF, or ascorbic acid, and a reduction in the FBS concentration lower than 7% led to gradual differentiation of the obtained iPSC lines.

**Properties of doxycycline-independent vole cell lines**

The obtained cell lines grow as flatten colonies with tight intercellular contacts and clear colony edge resembling human PSCs (*Fig. 2B*). Cell colonies grew pushing out the feeder cells and attached to the plastic- like human ESCs/iPSCs, but not above the feeder as mESCs. The proliferation intensity of the common vole iPSC lines was comparable to that of human PSCs. It was found that supplementation of the medium with a ROCK inhibitor significantly enhances cell survival both during mechanical transfer (by capillary) or upon using TrypLE.

More than 70% of the cells in both iPSC lines exhibited the expected number of autosomes, equal to 50; X chromosome from *M. levis *and Y chromosome from* M. arvalis *(*Fig. 2C*).

**Fig. 3:**
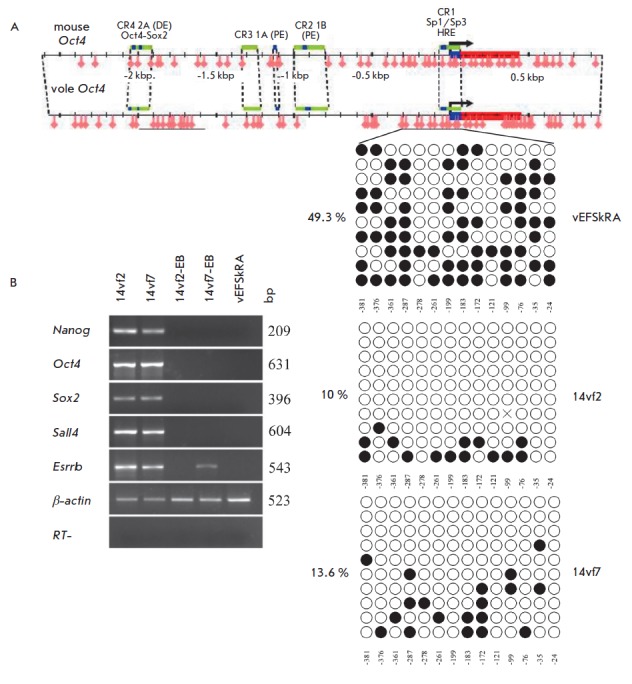
Analysis of CpG dinucleotide methylation in the *Oct4 *gene promoter and expression of genes specific for pluripotent cells in common vole iPSCs. *A ***– **comparison of CpG dinucleotide methylation of the *Oct4 *promoter in the obtained iPSC lines (14vf2, 14vf7) and initial line (vEFSkRA) of embryonic skin fibroblasts. Schematic distribution of CpG dinucleotides in a mouse and a common vole *Oct4 *promoter is presented at the top. Light and dark circles – unmethylated and methylated CpG dinucleotides, respectively. *B *– transcriptional activity of the genes responsible for the pluripotent state in the iPSC lines 14vf2 and 14vf7 and their differentiated derivatives (14vf2-EB and 14vf7-EB). Control – initial cell line vEFSkRA. (RT-) – negative control of reverse transcription reaction

A histochemical analysis demonstrated AP activity in undifferentiated cell lines (*Fig. 2D*), which was not detected after differentiation. Unlike in doxycyclinedependent lines, transcription of the introduced construct carrying reprogramming factors was not detected in stable iPSC lines of *M. levis *× *M. arvalis *hybrids (*Fig. 2E*). Moreover, both cell lines exhibited demethylation of CpG dinucleotides in the promoter of vole *Oct4*, which is indicative of its reactivation (*Fig. 3A*).

An immunofluorescence analysis showed that in undifferentiated state in the early passages the obtained cell lines expressed one of the key markers of pluripotency – surface antigen SSEA1 (*Fig. 2F*), which is specific to mESCs/iPSCs. *Fig. 2F* clearly shows that staining both for AP and SSEA1 is located in the most bulky, tight undifferentiated part of the colony, while the upper left edge of the colony, which contains spread differentiated cells, is not stained either for AP or for SSEA1. However, after a series of passages we failed to detect SSEA1-expressing cells. In *M. levis *×* M. arvalis *hybrid iPSCs, we also detected the expression of the key genes of pluripotent state *Oct4 *and* Sox2*, which remained stable during cell growth (*Fig. 2G*).

In both lines of *M. levis *× *M. arvalis *hybrid iPSCs the method of RT-PCR revealed expression of the endogenous genes *Nanog, Oct4, Sox2, Sall4, *and *Esrrβ*, which are essential for maintaining the pluripotent state in mammalian cells. The initial line of embryonic skin cells lacked transcripts of these genes (*Fig. 3B*). Thus, taking into account the PSC-like morphology of the cell colony, AP expression, demethylation of the* Oct4 *promoter, expression of *Nanog*, *Oct4*, *Sox2*, *Sall4,* and *Esrrb, *and unlimited proliferation in the absence of doxycycline in the culture without change in cell morphology, we can claim that we managed to obtain pluripotent iPSCs of *M. levis *× *M. arvalis *interspecific hybrids.

**Fig. 4:**
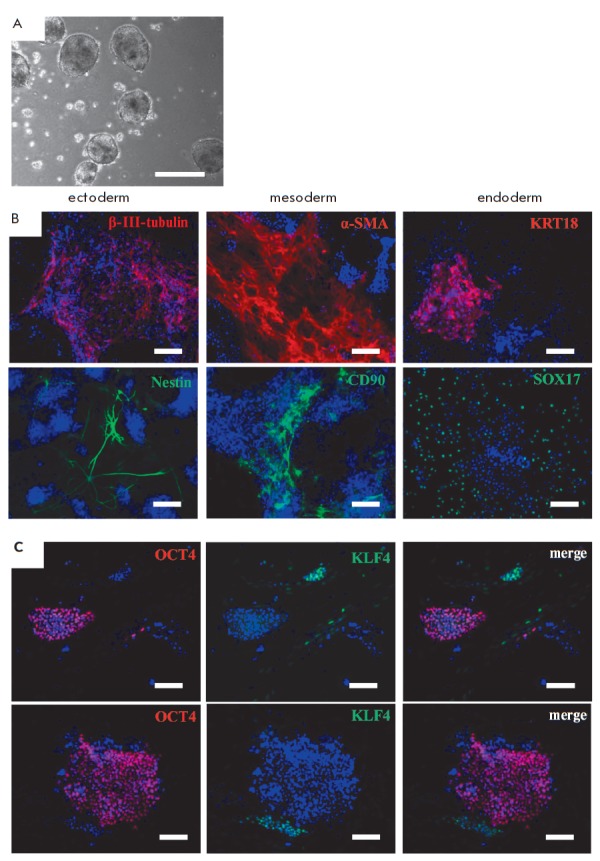
Spontaneous differentiation of common vole iPSCs. *A *– morphology of embryoid bodies formed from 14vf2 cells in suspension culture in 5 days. *B *– immunofluorescence analysis of differentiated derivatives of common vole iPSCs. Identification of ectoderm markers: β-III-tubulin (red signal), Nestin (green signal); mesoderm: α-SMA (red signal), CD90 (green signal); endoderm: KRT18 (red signal), SOX17 (green signal). *C ***– **immunofluorescent detection of the transcription factors OCT4 (red signal) and KLF4 (green signal). Nuclei are stained with DAPI (blue signal). Scale bar *A *– 500 μm,* B, C *– 100 μm

In order to study the ability of the cell lines to differentiate* in vitro*, we obtained embryoid bodies, which had already formed in the suspension culture by day 2 (*Fig. 4A*). Analysis of differentiated derivatives by immunofluorescent staining with antibodies to markers of specific cell types revealed derivatives in all three primary germ layers: ectoderm (Nestin, β-III-tubulin), endoderm (SOX17, KRT18), and mesoderm (α-SMA, CD90) (*Fig. 4B*).

Investigating the spontaneous differentiation of* M. levis *× *M. arvalis *hybrid iPSCs that have undergone successful reprogramming in culture using antibodies that detect only endogenous OCT4 and KLF4, we found that expression of KLF4 is absent in pluripotent cells, but it appears at the beginning of their differentiation, correlating with the loss of the transcription factor OCT4 (*Fig. 4C*).

## DISCUSSION

We have attempted to obtain iPSCs of hybrids from crossing *M. levis *and *M. arvalis *species, which belong to a group of common voles, genus *Microtus*. The first experiments on obtaining common vole iPSCs were performed using the same culture media and factors that had previously allowed to induce pluripotency and obtain iPSCs of prairie vole *M. ochrogaster *[16]. Prairie vole iPSCs were obtained in the presence of a KSR substitute for fetal bovine serum, as well as LIF cytokine, which triggers a key signaling cascade in mouse and rat pluripotent cells [57-59]. Generation of* M. ochrogaster *iPSCs can be achieved in the presence of the inhibitors CHIR99021, PD0325901, and A83-01 in the medium. Nevertheless, the induction of pluripotency did not take place upon reprogramming of common vole cells using four OSKM factors and a medium containing LIF and KSR. After careful selection of conditions, hybrid *M. levis *× *M. arvalis *iPSCs were obtained in a medium containing mLIF, bFGF, ascorbic acid, and a mixture of 7% FBS and 7% KSR. It is interesting that in most cases could not we obtain even primary iPSC-like colonies during the reprogramming of *M. ochrogaster *cells in a medium containing bFGF and FBS.

Our experience shows that mouse OSKM, which is used for the induction of pluripotency in somatic cells of different types and in different species, can be effective in obtaining common vole iPSCs. However, the presence of LIF cytokine in the medium, an important factor in the formation and maintenance of undifferentiated state in ESCs and iPSCs in rodents (mouse, rat, as well as prairie vole) [16, 57-59], is not sufficient for the induction and maintenance of pluripotency in common vole cells *in vitro*. This result is consistent with the reports of unsuccessful attempts to obtain ESCs of common vole group species from the inner cell mass of blastocysts in the presence of mouse or *M. levis *LIF [31]. Taken together, these data allow one to suggest that the signaling pathway triggered by cytokine LIF, due to some species-specific features of the common vole, cannot independently provide pluripotency in *in vitro* conditions.

The factors bFGF and mLIF comprised the key combination for the induction and maintenance of pluripotency in common vole cells. Factor bFGF is known to participate in the triggering of the main signaling cascade of PSCs in such species as human, monkey, dog, cow, horse, and sheep [7, 9, 13, 15, 17, 51-55, 60-62]. A combination of LIF and bFGF is also used for the induction and maintenance of pluripotency in many mammalian species, including human [63], rabbit [13], dog [62], horse [7, 17], and sheep [9]. Comparison of human PSCs obtained and cultured in different conditions one of which includs bFGF only, and another bFGF, and LIF together showed that iPSCs and ESCs maintained using two factors are more similar in their characteristics of the transcriptome and epigenome to pluripotent cells of early embryos [63].

An important medium component that allowed us to conduct the reprogramming of differentiated cells of common voles to a pluripotent state is ascorbic acid. It has previously been shown that ascorbic acid possesses antioxidant properties, as well as activates histone demethylases and the TET proteins responsible for the most important epigenetic transformations upon pluripotency induction, which, in particular, is essential for the initiation of endogenous *Oct4 *and *Nanog *expression in reprogrammed cells [47, 49 ].

It has been demonstrated on many mammalian species that it is preferable to use a medium supplemented with KSR, not FBS, when working with pluripotent cells [10, 13, 15, 16, 60]. However, during reprogramming of common vole fibroblasts to a pluripotent state, primary cell colonies were only obtained in media containing not less than 7% FBS. Moreover, substitution of FBS with KSR in the culture medium induced differentiation of the obtained common vole iPSCs even in the presence of growth factors.

The presence of mLIF, bFGF, ascorbic acid, and FBS in the culture medium is essential for maintaining the self-renewal and pluripotency capabilities of common vole iPSCs. Removal of any of these components triggers differentiation.

The obtained stable common vole iPSC lines share the properties of pluripotent cells. They exhibit AP activity and express the endogenous transcription factors* Nanog, Oct4, Sox2, Esrrb*, and *Sall4*, which are necessary for maintaining PSCs in the undifferentiated state [64-68]. Hereupon, the promoter region of* Oct4 *is more hypomethylated in iPSCs than in embryonic fibroblasts. The obtained lines of common vole pluripotent cells are capable of unlimited selfrenewal and differentiation *in vitro *into derivatives of the three primary germ layers. Nevertheless, unlike* M. ochrogaster *iPSCs, stable cell lines of common voles with induced pluripotency do not maintain SSEA1 expression during culturing, and they also do not express KLF4. Probably, this may be due to the fact that various signaling cascades participate in maintaining the pluripotency of *M. ochrogaster *and common vole iPSCs. The expression of KLF4 and SSEA1 is specific to PSCs in which a key signaling cascade providing pluripotency is triggered by cytokine LIF and varies or absent in PSCs, the pluripotency of which is supported by a bFGF-activated signaling cascade [61, 69- 71]. Thus, pluripotent cell lines derived from postimplantation mouse embryos in the presence of bFGF do not express KLF4, unlike ESCs and iPSCs cultured with LIF [71, 72]. Sheep, macaque, and human PSCs obtained in the presence of bFGF do not exhibit the SSEA1 surface antigen [8, 15, 61, 69]. It is noted that the pluripotent cells maintained with bFGF exhibit flatter morphology and proliferate slower [8, 72]. These two properties of bFGF-dependent pluripotent cells are specific to common vole iPSC lines. To speculate on whether the obtained iPSCs of *M. ochrogaster* and common voles fully reflect the properties of pluripotent cells of these species appears impossible: ESC lines that serve as a standard of pluripotency are absent both in prairie and common vole cultures. Nothing is known either about the properties of pluripotent cells of the pre- and postimplantation embryos of these species *in vivo*.

Thus, the technology of reprogramming by overexpression of four OSKM transcription factors allowed us to obtain for the first time common vole iPSCs, pluripotent cell lines that had been previously impossible to grow as a culture. These lines are to be used to study the processes of early development and pluripotency genes in common voles. We hope that the experience gained in the course of this work will allow us to further develop more effective approaches for the reprogramming of somatic cells of common voles and isolate ESCs from early preimplantation blastocysts and germinal cells of these rodents.

## CONCLUSIONS

In this study, iPSCs of common vole *M. levis *× *M. arvalis* hybrids were obtained in a medium containing cytokine LIF, bFGF, ascorbic acid, and FBS. Common vole iPSCs obtained in these conditions are capable of self-renewal, they express the pluripotency genes *Oct4, Nanog, Sox2, Sall4, *and *Esrrb *and form derivatives of the three primary germ layers during differentiation *in vitro*. The results of our work will allow researchers to assess diversity and species-specific features in induction and maintenance of the pluripotent cells of various mammalian species.

## Abbreviations

iPSCs: induced pluripotent stem cells
mESCs: mouse embryonic stem cells
PSCs: pluripotent stem cells
ESCs: embryonic stem cells
AP: alkaline phosphatase
OSKM: transcription factors Oct4, Sox2, Klf4 and c-Myc
RT-PCR: reverse transcription followed by polymerase chain reaction

## References

[1] Evans M.J., Kaufman M.H. (1981). Nature.

[2] Martin G.R. (1981). Proc. Natl. Acad. Sci. USA..

[3] Takahashi K., Yamanaka S. (2006). Cell..

[4] Dutta D. (2013). Int. J. Dev. Biol..

[5] Medvedev S.P., Shevchenko A.I., Elisaphenko E.A., Nesterova T.B., Brockdorff N., Zakian S.M. (2008). BMC Genomics..

[6] Medvedev S.P., Elisaphenko E.A., Mazurok N.A., Zakian S.M. (2009). Dokl. Biochem. Biophys...

[7] Breton A., Sharma R., Diaz A.C., Parham A.G., Graham A., Neil C., Whitelaw C.B., Milne E., Donadeu F.X. (2013). Stem Cells Dev..

[8] Li Y., Cang M., Lee A.S., Zhang K., Liu D. (2011). PLoS One..

[9] Liu J., Balehosur D., Murray B., Kelly J.M., Sumer H., Verma P.J. (2012). Theriogenology..

[10] Wu Z., Chen J., Ren J., Bao L., Liao J., Cui C., Rao L., Li H., Gu Y., Dai H. (2009). J. Mol. Cell Biol..

[11] Bao L., He L., Chen J., Wu Z., Liao J., Rao L., Ren J., Li H., Zhu H., Qian L. (2011). Cell Res..

[12] Ezashi T., Telugu B.P., Alexenko A.P., Sachdev S., Sinha S., Roberts R.M. (2009). Proc. Natl. Acad. Sci. USA..

[13] Honda A., Hirose M., Hatori M., Matoba S., Miyoshi H., Inoue K., Ogura A. (2010). J. Biol. Chem..

[14] Liao J., Cui C., Chen S., Ren J., Chen J., Gao Y., Li H., Jia N., Cheng L., Xiao H. (2009). Cell Stem Cell..

[15] Liu H., Zhu F., Yong J., Zhang P., Hou P., Li H., Jiang W., Cai J., Liu M., Cui K. (2008). Cell Stem Cell..

[16] Manoli D.S., Subramanyam D., Carey C., Sudin E., van Westerhuyzen J.A., Bales K.L., Blelloch R., Shah N.M. (2012). PLoS One..

[17] Nagy K., Sung H.K., Zhang P., Laflamme S., Vincent P., Agha-Mohammadi S., Woltjen K., Monetti C., Michael I.P., Smith L.C. (2011). Stem Cell Rev..

[18] Shimada H., Nakada A., Hashimoto Y., Shigeno K., Shionoya Y., Nakamura T. (2010). Mol. Reprod. Dev..

[19] Takahashi K., Tanabe K., Ohnuki M., Narita M., Ichisaka T., Tomoda K., Yamanaka S. (2007). Cell..

[20] Mazurok N.A., Rubtsova N.V., Isaenko A.A., Pavlova M.E., Slobodyanyuk S.Y., Nesterova T.B., Zakian S.M. (2001). Chromosome Res..

[21] Dementyeva E.V., Shevchenko A.I., Anopriyenko O.V., Mazurok N.A., Elisaphenko E.A., Nesterova T.B., Brockdorff N., Zakian S.M. (2010). Chromosoma..

[22] Rubtsov N.B., Rubtsova N.V., Anopriyenko O.V., Karamysheva T.V., Shevchenko A.I., Mazurok N.A., Nesterova T.B., Zakian S.M. (2002). Cytogenet. Genome Res..

[23] Shevchenko A.I., Malakhova A.A., Elisaphenko E.A., Mazurok N.A., Nesterova T.B., Brockdorff N., Zakian S.M. (2011). PLoS One..

[24] Shevchenko A.T., Mazurok N.A., Slobodyanyuk S.Y., Zakian S.M. (2002). Chromosome Res..

[25] Vaskova E.A., Dementyeva E.V., Shevchenko A.I., Pavlova S.V., Grigor’eva E.V., Zhelezova A.I., Vandeberg J.L., Zakian S.M. (2014). PLoS One..

[26] Sherstyuk V.V., Shevchenko A.I., Mazurok N.A., Zakian S.M. (2013). Dokl. Biochem. Biophys..

[27] Nesterova T.B., Slobodyanyuk S.Y., Elisaphenko E.A., Shevchenko A.I., Johnston C., Pavlova M.E., Rogozin I.B., Kolesnikov N.N., Brockdorff N., Zakian S.M. (2001). Genome Res..

[28] Zakian S.M., Kulbakina N.A., Meyer M.N., Semenova L.A., Bochkarev M.N., Radjabli S.I., Serov O.L. (1987). Genet. Res..

[29] Sorokin M.A., Medvedev S.P., Shevchenko A.I., Slyn’ko N.M., Zakiian S.M. (2010). Rus. J. Genet..

[30] Mazurok N.A., Rubtsova N.V., Grigor’eva E.V., Matveeva N.M., Zhelezova A.I., Shilov A.G., Slobodianiuk S., Zakian S.M. (2003). Rus. J. Dev. Biol..

[31] Grigor’eva E.V., Shevchenko A.I., Mazurok N.A., Elisaphenko E.A., Zhelezova A.I., Shilov A.G., Dyban P.A., Dyban A.P., Noniashvili E.M., Slobodyanyuk S.Y. (2009). PLoS One..

[32] Shevchenko A.I., Demina V.V., Mazurok N.A., Zhelezova A.I., Efremov Ia R., Shilov A.G., Shevela A.I., Belevantseva A.V., Vlasov V.V., Zakiian S.M. (2008). Rus. J. Genet..

[33] Grigor’eva E.V., Shevchenko A.I., Zhelezova A.I., Shilov A.G., Mazurok N.A., Dyban P.A., Dyban A.P., Zakian S.M. (2011). Bull. Exp. Biol. Med..

[34] Carey B.W., Markoulaki S., Hanna J., Saha K., Gao Q., Mitalipova M., Jaenisch R. (2009). Proc. Natl. Acad. Sci. USA..

[35] Maherali N., Ahfeldt T., Rigamonti A., Utikal J., Cowan C., Hochedlinger K. (2008). Cell Stem Cell..

[36] Kingston R.E., Chen C.A., Okayama H. (2003). Curr. Protoc. Cell Biol. 2003. Chapter 9. Unit 9.1..

[37] Pain B., Clark M.E., Shen M., Nakazawa H., Sakurai M., Samarut J., Etches R.J. (1996). Development..

[38] Kumaki Y., Oda M., Okano M. (2008). Nucleic Acids Research.

[39] Nesterova T.B., Duthie S.M., Mazurok N.A., Isaenko A.A., Rubtsova N.V., Zakian S.M., Brockdorff N. (1998). Chromosome Res..

[40] Sorokin M.A., Elisafenko E.A., Mazurok N.A., Zakian S.M. (2013). Dokl. Biochem. Biophys..

[41] Bechard M., Dalton S. (2009). Mol. Cell. Biol..

[42] Buehr M., Meek S., Blair K., Yang J., Ure J., Silva J., McLay R., Hall J., Ying Q.L., Smith A. (2008). Cell..

[43] Chang M.Y., Kim D., Kim C.H., Kang H.C., Yang E., Moon J.I., Ko S., Park J., Park K.S., Lee K.A. (2010). PLoS One..

[44] Chen Y., Blair K., Smith A. (2013). Stem Cell Reports..

[45] Li P., Tong C., Mehrian-Shai R., Jia L., Wu N., Yan Y., Maxson R.E., Schulze E.N., Song H., Hsieh C.L. (2008). Cell..

[46] Ying Q.L., Wray J., Nichols J., Batlle-Morera L., Doble B., Woodgett J., Cohen P., Smith A. (2008). Nature.

[47] Blaschke K., Ebata K.T., Karimi M.M., Zepeda-Martinez J.A., Goyal P., Mahapatra S., Tam A., Laird D.J., Hirst M., Rao A. (2013). Nature.

[48] Dutta D., Ray S., Home P., Larson M., Wolfe M.W., Paul S. (2011). Stem Cells..

[49] Esteban M.A., Wang T., Qin B., Yang J., Qin D., Cai J., Li W., Weng Z., Chen J., Ni S. (2010). Cell Stem Cell..

[50] Rajendran G., Dutta D., Hong J., Paul A., Saha B., Mahato B., Ray S., Home P., Ganguly A., Weiss M.L. (2013). J. Biol. Chem..

[51] Medvedev S.P., Grigor’eva E.V., Shevchenko A.I., Malakhova A.A., Dementyeva E.V., Shilov A.A., Pokushalov E.A., Zaidman A.M., Aleksandrova M.A., Plotnikov E.Y. (2011). Stem Cells Dev..

[52] Medvedev S.P., Malakhova A.A., Grigor’eva E.V., Shevchenko A.I., Dementyeva E.V., Sobolev I.A., Lebedev I.N., Shilov A.G., Zhimulev I.F., Zakian S.M. (2010). Acta Naturae..

[53] Medvedev S.P., Shevchenko A.I., Zakian S.M. (2010). Acta Naturae..

[54] Medvedev S.P., Shevchenko A.I., Zakian S.M. (2010). Acta Naturae..

[55] Shutova M.V., Bogomazova A.N., Lagarkova M.A., Kiselev S.L. (2009). Acta Naturae..

[56] Ginis I., Luo Y., Miura T., Thies S., Brandenberger R., Gerecht-Nir S., Amit M., Hoke A., Carpenter M.K., Itskovitz-Eldor J. (2004). Developmental Biology.

[57] Vassilieva S., Guan K., Pich U., Wobus A.M. (2000). Exp. Cell Res..

[58] Smith A.G.., Heath J.K., Donaldson D.D., Wong G.G., Moreau J., Stahl M., Rogers D. (1988). Nature.

[59] Williams R.L., Hilton D.J., Pease S., Willson T.A., Stewart C.L., Gearing D.P., Wagner E.F., Metcalf D., Nicola N.A., Gough N.M. (1988). Nature.

[60] Han X., Han J., Ding F., Cao S., Lim S.S., Dai Y., Zhang R., Zhang Y., Lim B., Li N. (2011). Cell Res..

[61] Thomson J.A., Itskovitz-Eldor J., Shapiro S.S., Waknitz M.A., Swiergiel J.J., Marshall V.S., Jones J.M. (1998). Science..

[62] Vaags A.K., Rosic-Kablar S., Gartley C.J., Zheng Y.Z., Chesney A., Villagomez D.A., Kruth S.A., Hough M.R. (2009). Stem Cells..

[63] Gafni O., Weinberger L., Mansour A.A., Manor Y.S., Chomsky E., Ben-Yosef D., Kalma Y., Viukov S., Maza I., Zviran A. (2013). Nature.

[64] Chambers I., Colby D., Robertson M., Nichols J., Lee S., Tweedie S., Smith A. (2003). Cell..

[65] Chambers I., Silva J., Colby D., Nichols J., Nijmeijer B., Robertson M., Vrana J., Jones K., Grotewold L., Smith A. (2007). Nature.

[66] Masui S., Nakatake Y., Toyooka Y., Shimosato D., Yagi R., Takahashi K., Okochi H., Okuda A., Matoba R., Sharov A.A. (2007). Nat. Cell. Biol..

[67] Mitsui K., Tokuzawa Y., Itoh H., Segawa K., Murakami M., Takahashi K., Maruyama M., Maeda M., Yamanaka S. (2003). Cell..

[68] Niwa H., Miyazaki J., Smith A.G. (2000). Nat. Genet..

[69] Adewumi O., Aflatoonian B., Ahrlund-Richter L., Amit M., Andrews P.W., Beighton G., Bello P.A., Benvenisty N., Berry L.S., Bevan S. (2007). Nat. Biotechnol..

[70] Osorno R., Chambers I. (2011). Philos. Trans. R. Soc. Lond. B Biol. Sci..

[71] Posfai E., Tam O.H., Rossant J. (2014). Curr. Top. Dev. Biol..

[72] Tesar P.J., Chenoweth J.G., Brook F.A., Davies T.J., Evans E.P., Mack D.L., Gardner R.L., McKay R.D. (2007). Nature.

